# Improving Urban Water Environment in Eastern China by Blending Traditional with Modern Landscape Planning

**DOI:** 10.1155/2017/6967145

**Published:** 2017-03-12

**Authors:** Jiajie Cao, Junjun Yu, Yuan Tian, Cai Zhao, Hao Wang

**Affiliations:** College of Landscape Architecture, Nanjing Forestry University, Nanjing 210037, China

## Abstract

As a fundamental part of greenspace, urban water landscape contributes greatly to the ecological system and at the same time supplies a leisure area for residents. The paper did an analysis on the number of aquatic plant communities, the form of water spaces, and water quality condition by investigating 135 quadrats (90 at amphibious boundary and the land, 45 in the water) in 45 transects of 15 urban and suburban parks. We found that water spaces had monotonous forms with low biodiversity and poor water quality. In addition, urban water landscapes hardly provided ecological functions given excessive construction. Accordingly, a proposition to connect tradition with modernism in the improvement and innovation of urban water landscape planning was put forward, and further, the way to achieve it was explored. By taking Qinhu Wetland Park as a case, the principles and specific planning methods on macro- and microperspectives were discussed to guide the development of urban landscape in eastern China.

## 1. Background: The Condition of Urban Water Landscape in China

According to “2015 Report on the State of the Environment of China,” the result of surface water quality monitoring in 967 sites distributed in 423 main rivers and 62 lakes and reservoirs showed that water quality in I*～*III category, IV*～*V category, and V category accounted for 64.5%, 26.7%, and 8.8% respectively. Since the 13th five year plan, Chinese government has been investing heavily on water environment treatment. For example, Wuxi city plans to put 6 billion RMB on more than 26 rivers including the ancient canal, Huancheng river, the grand canal, and Liangxi river to build urban water tourism landscape; Baotou city invested over 18 billion RMB in the improvement and utilization of urban water ecosystem; Shantou city invested around 10.8 million RMB to treat black and foul water. However, serious conditions of water environment still remain although great achievements of water environment treatment have been made compared with the past.

Located in the middle and lower reaches of the Yangtze, Nanjing is a city with rich water resources. Four water systems can be divided as the Yangtze, Chu River, Qinhuai River, and Shuiyang River, including 564 rivers in total among which 57 rivers cover more than 50 km^2^. In the 1950s, the amount of lakes and ponds in urban area reached over 300. However, it decreased dramatically since then: Xuanwu lake, Yanque lake, and Mochou lake have degenerated into inland lakes after rounds of landfill; few water landscapes were left and lots of ponds and lakes became none-penetrated surface destructive to water systems as well as urban geological landscape at the end of 1990s. Nowadays, most water landscapes in Nanjing belong to closed or illiquid small systems that get easily polluted with low water environmental capacity and poor self-purification capability [1].

At present, two aspects are worth considering in water environment treatment. Firstly, the construction and reform of urban water system in close touch with people need to be taken as seriously as the treatment of large rivers and lakes. Secondly, how to construct water landscape in a scientific way should be considered given the shortage of relative researches and standards [2]. In this case, this paper investigated means to build attractive landscape with sustainability in urban areas based on the perspective of ecological restoration and aesthetic functions of landscape.

## 2. Conditions of Urban Water Landscape in Nanjing

### 2.1. Research Location

Located in the middle and lower reaches of the Yangtze, Nanjing is a land mainly with low mountains and gradual hills. Rich in the water resources (learned from “Nanjing water introduction”); it owns altogether 564 rivers with average volume of 1.8 billion m^3^, among which surface water resources reach up to 22.1 billion m^3^.

**Figure 1:**
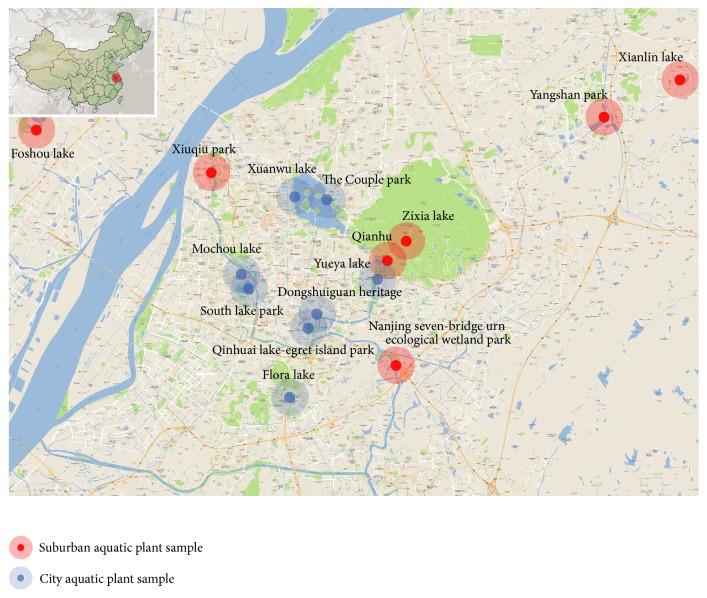
Fifteen research sites in Nanjing.

We collected data on 15 wetland parks in Nanjing (Figure 1, 8 parks in the urban area and 7 parks in the suburbs). Three urban wetland parks as Qinhuai River, Xuanwu lake, and Mochou lake belong to main river systems in Nanjing. 15 plots are as follows: Xuanwu lake (Xuanwu men), the Couple park, Xianlin lake, Yueya lake, Qinhuai lakeegret island park, Yangshan park, Nanjing seven-bridge urn ecological wetland park, Jiulong lake, Foshou lake, Mochou lake, Xiuqiu park, Dongshuiguan heritage (Qinhuai lake), and Qianhu.

### 2.2. Methods and Results

**Figure 2:**
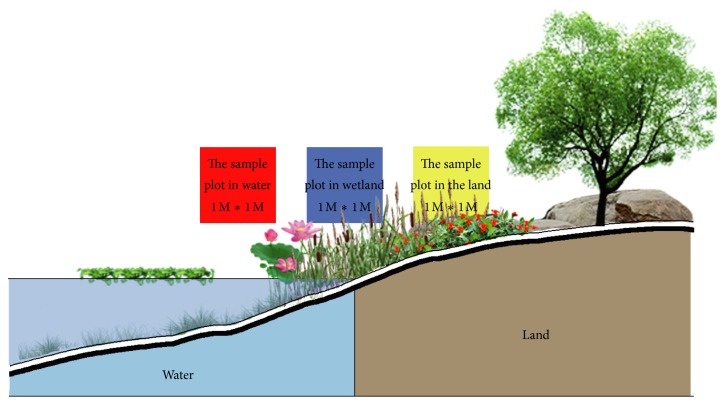
Nanjing seven-bridge urn ecological wetland park aquatic plant status (local).

We used typical sample method to collect data. Three transects (1 meter wide and 4 meters long) perpendicular to the amphibious boundaries were arranged in each location. In each transect, three quadrats at 1 m × 1 m were set up at the water, amphibious boundary and the land, respectively (Figure 2). The interval distance between each quadrat varied from 0.5 to 1 m depending on the specific landform; for example, quadrats at the two ends of a clear amphibious boundary could connect. Quadrats in the water extended to the threshold covered by water vascular plants, while those in amphibious boundary and the land were arranged on moisture gradient until the appearance of massive typical xerophytia or road edge. Altogether 135 quadrats (90 at amphibious boundary and the land, 45 in the water) were arranged in 45 transects of 15 urban and suburban parks.

Survey in position was used to make records on the number of aquatic plant species, their frequency, and plant coverage (the proportion of area covered by crown geometry shade to that of the quadrats) sorted by importance calculations (relative plant coverage as the coverage of one species to the area covered by all species). After 15 days, data in 45 transects were collected.  Table 1 showed the longitude and latitude of one transect as an example at each location.

The research showed that sixty species were usually seen in Nanjing wetland parks: 3 as submerged plants, 4 as floating-leaved plants, 12 as emerging plants, and 41 as hygrophytes. In the suburbs, we collected 23 aquatic species in total, among which 2 were submerged plants, 2 floating-leaved plants, 14 hygrophytes, and 5 emerging plants (Table 2). Deficiency in species accompanied by wide distribution of certain kinds was universal in suburban parks.* Persicaria lapathifolia* reached 81% in frequency and ranked the first in all species. In the species of hygrophytes,* Ranunculus sceleratus* and* Alternanthera philoxeroides* also showed high frequency at 57%. The frequency of* Potamogeton crispus* was up to 43% in submerged plants.* Iris tectorum* and* Arundinella anomala* showed the highest frequency in emerging plants at 38% (the same with Spirogyra in floating-leaved plants).* Potamogeton crispus* ranked the highest in relative coverage at 0.95 in all species, followed by* Spirogyra* at 0.68,* Iris tectorum* at 0.41,* Arundinella anomala* at 0.32,* Lemna minor* at 0.32,* Persicaria lapathifolia* at 0.2, and* Phragmites australis* at 0.19.

Table 3 showed that 26 species could be found in central city parks.* Persicaria lapathifolia* also ranked the highest in frequency at 79.2% in all species, followed by* Alternanthera philoxeroides* at 66.7%,* reeds* at 63.4%,* Potamogeton crispus* at 58.3%,* Spirogyra* at 58.3%, and* Iris tectorum* at 50%.* Potamogeton crispus* ranked the highest in relative coverage at 0.93 in all species, followed by* Iris tectorum* at 0.48,* Spirogyra* at 0.43,* Lemna minor* at 0.38, and* Phragmites australis* at 0.34;* Persicaria lapathifolia* at 0.22,* Nymphaea tetragona*,and* Alternanthera philoxeroides* at 0.19.

**Figure 3:**
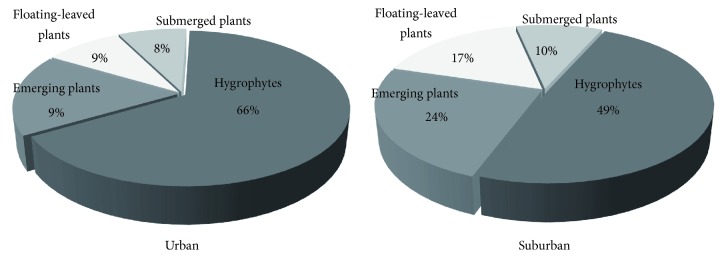
Percentages of aquatic plant life form urban and suburban parks.

As it is shown in Figure 3, both urban and suburban parks had a tendency that hygrophytes and emerging plants were widely distributed, but much less submerged and floating-leaved plants appeared. In addition, aquatic plants were concentrated on certain types as* Persicaria lapathifolia*,* Alternanthera philoxeroides*,* Spirogyra*,* Potamogeton crispus*, and* Iris tectorum*.

**Figure 4:**
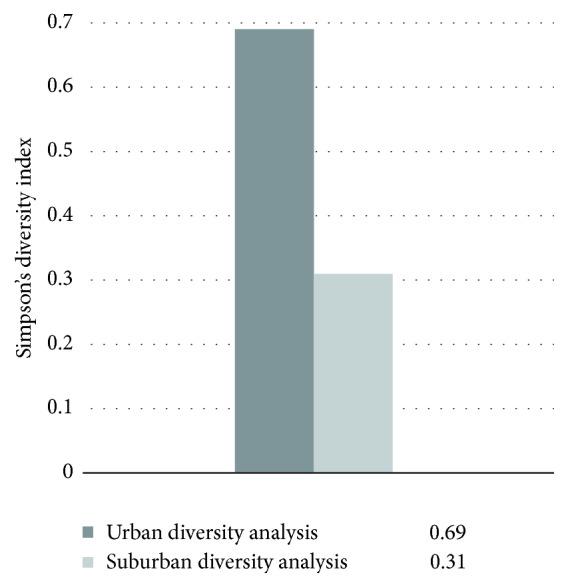
Urban and suburban diversity analysis.

Comparatively speaking, plants showed higher Simpson diversity in urban parks than those in the suburbs (Figure 4). Comparing with urban parks, suburban parks show a decrease in the proportion of hygrophytes with growing number of submerged and floating-leaved plants (Figure 3). Specifically, Simpson diversity was the lowest in Huashen lake and Foshou lake parks in the suburbs, while it reached the highest in Dongshuiguan heritage at 0.65 in central city.

### 2.3. Analysis and Suggestions

In this paper, we made a survey on the plants of urban and suburban wetland parks in Nanjing. Main findings are as follows.

Firstly, diversity of aquatic plants needs to be enhanced through human intervention. Generally speaking, the results showed that Simpson diversity in urban parks was higher than those in the suburbs. It may greatly be attributed to the government policies advocating plant configuration and maintenance in urban parks. However, limited choices of aquatic plants in landscape building are a problem. High frequency of certain species brings negative effects to ecological functions. In addition, the weak vertical stratification resulting from unbalanced proportion of hygrophytes, emerging plants, submerged plants, and floating-leaved plants can hardly bring aesthetic experiences to the audience.

Second, considering the ecological and aesthetic functions of wetlands, frequency of plant species needs to be taken into account with plant coverage in wetland management. The research showed that although* Potamogeton crispus* did not show most frequently, it ranked the first in relative coverage both in urban and in suburban parks. On the contrary, the most frequently shown plant,* Persicaria lapathifolia* lagged far behind in relative coverage (0.22 in urban parks and 0.2 in suburban parks). On the aspect of ecological functions, high relative coverage of some species may violate the habitats of other plants. As for wetland landscape planning, large scale coverage of certain plants would definitely cause fatigue for the audience by their excessive appearances.

### 2.4. Urban Water Landscape Planning: Tradition and Developments

Researches have been supporting the great influences of greenspace on ecological performance [3], among which water landscape works as a fundamental part. However, overcommercialization lack of scientific planning and maintenance is destroying the quality of urban water landscape and further weakening the ecological and economic benefits brought by water systems. Traditional landscape planning deals with such problems mainly in the following parts.

*Landscape Layouts.* Scholars proposed topography and geomorphy plans adapted to natural forms and spatial patterns in a specific ecosystem. For example, a streamline shape is promoted for curved waterline contributes to protect ecological diversity by providing habitats with rich resources [4].

*Landscape Architecture.* Architectures in a landscape are necessary to meet people's needs of sightseeing and recreation. Generally speaking, large scale buildings should be placed away from the location of the best view, while those in small scale can be built in the scenic beauty spots in a style coordinated with the whole water landscape. Architectures submerged in the water can cover a small space in “bottom elevated” shape so as to reduce the disturbing effect to ecosystems. It is preferable to use natural materials or concrete construction of trunks and stones in landscape buildings [5–7].

*Revetments.* Ecological revetments, especially those varied with shore side situations, are preferred, such as ecological friendly planted revetments or lifted natural matrix soil instead of hard bulkhead to fulfil transition from land to lakes. Landlake ecozone should be in a stretched natural curve so as to embrace various habitats for animals [8, 9].

*Plants.* Generally speaking, submerged plants, floating and floating-leaved plants, emerging plants, and hygrophyte (preferably local species) are used in water landscape. Choices should be adjusted to meet plants' living habits in a consideration of biodiversity [10–12].

However, scholars have proposed critics towards these commonly used methods for their unsatisfactory management of plant diversity and the richness of layouts. For example, Freedman et al. (2006) pointed out that limited kinds of aquatic plants were applied in a monotonous layouts in urban wetland landscape. As it was shown in the findings of Nanjing urban wetland research, diversity both in species and in the spatial form of wetlands was in urgent need to be enhanced. Considering this, we introduce a successful case of urban water landscape planning in Eastern China to explore possible ways in wetland management innovation.

## 3. Case of Qinhu National Wetland Park: A Connection of Tradition and Modernism

**Figure 5:**
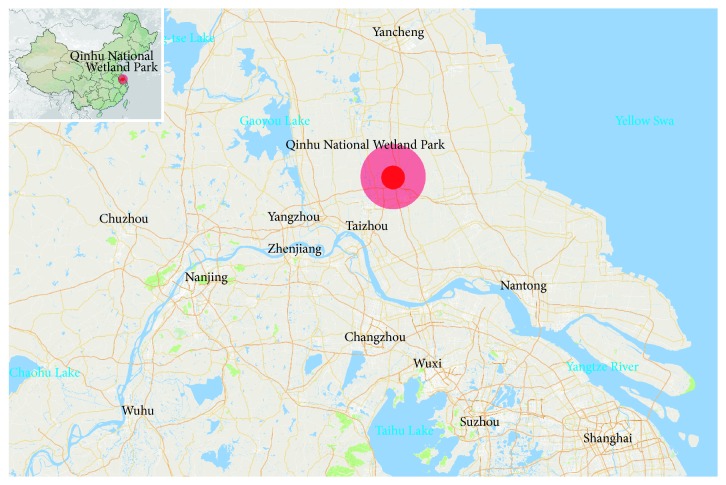
The location map of Qinhu National Wetland Park.

In a northern subtropical monsoon climate, Qianhu National Wetland Park is located in the south of Lixia river basin, one of the most three famous basins in China. As a national 5A level urban wetland park, it owns significant wetland resources in the middle and lower reaches of the Yangtze and in central Jiangsu province (Figure 5). The fresh water wetland of Qinhu is rarely seen in China, among which natural lake wetlands account for 10.68 sq.km and man-made wetlands occupy 32.61 sq.km with various types as moor, lakes, rivers, and man-made wetlands [13].

**Figure 6:**
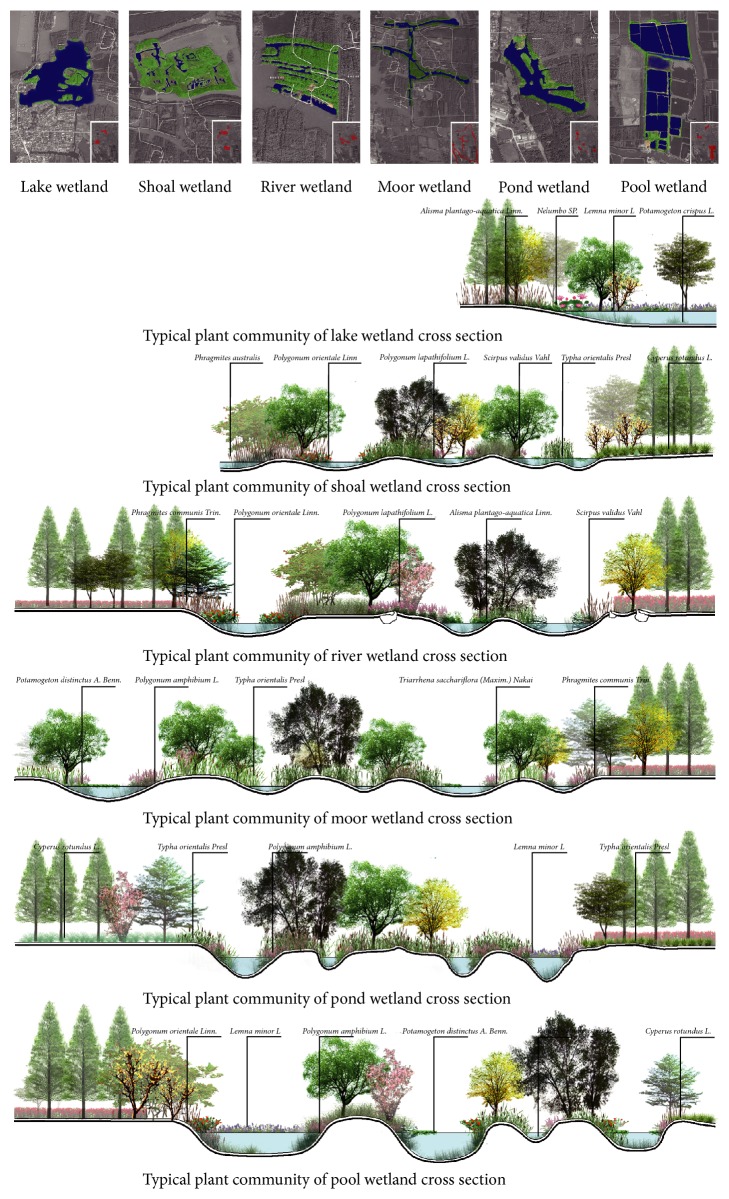
Plant community of wetland cross section.

Since the mid-20th century, due to land scarcity brought by population growth, excessive utilization on Qinhu wetland to reclaim land from lakes has greatly destroyed wetland ecology by turning large scale of wetlands to farms. With the awareness of wetland protection these days, in 2007, Qinhu National Wetland Park was listed in the protection network of the middle and lower reaches of the Yangtze with a conservation area of 26 sq.km (core area as 6 sq.km) and has been under continuous construction since then [14]. It provided the following enlightenments (Figure 6).

*(1) Enriching Spatial Forms in Water Landscape.* A multiscale spatial form was created to bring diverse wetland types: various landscape forms can be seen in Qinhu wetland, such as lakes, rivers, moors, mudflats, ponds, and pools, by making full use of existing situation.

In the lake wetlands occupying a large area, a flat terrestrial-aquatic transverse zone was designed to enlarge the contact area of land and water so that plants and animals would enjoy more living spaces. In the river wetlands, a terrestrial-aquatic transverse zone with varied illumination intensity could be seen to satisfy the demands of plant growth in a consideration of changing coastlines. In the moor and mudflat wetlands, plants instead of artificial factors were used to enrich wetland functions. In the pond and pool wetlands, a unique regional environment was created by embracing local plants, culture, and heritage into the planning.

**Figure 7:**
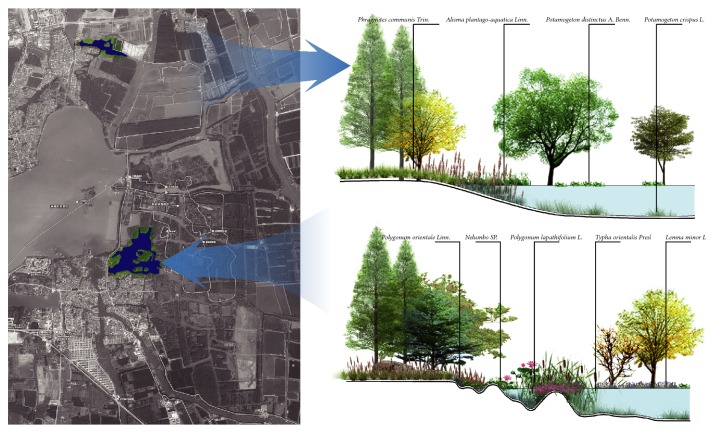
The structural comparison of revetments cross section.

*(2) Enriching the Cross Section Structure of Terrestrial-Aquatic Transverse Zones.* Transiting from water to land ecosystem, the terrestrial-aquatic transverse zone is significant in wetland landscape. Different from traditional ecological revetments design, diversified structures of cross section were created in Qinhu Wetland Park by constructing microtopography in the transverse zone. Meanwhile, varied kinds of plants were adopted to enrich spatial forms so as to build manifold habitats as well as coastline landscapes (Figure 7).

As a result, the ecology of Qinhu wetland has been greatly improved. With growing self-adjustment capacity, the biocenosis remained stable where biodiversity increased the preliminary statistics showing that there were 153 kinds of plants, 97 kinds of birds, 21 kinds of beasts, 38 kinds of fishes, and 21 kinds of floating animals. The main plants were as follows:* fluitantes as diatom*,* Cyanophyta*, and* chlorella*, and so forth; floating plants as* Eichhornia crassipes*,* Nymphaeales*,* Floral Aquatic*,* Salvinia natans*, and so forth; submerged plants as* Ceratophyllum demersum*,* Potamogeton distinctus*; water woody plants as* Taxodium ascendens*,* Metasequoia glyptostroboides*,* dryland willow*,* Pterocarya stenoptera*,* italian popular*, and so forth; water herbage as* Iris wilsonii*,* Typha orientalis*,* reed*,* Erigeron annuus*,* Indian Kalimeris Herb*,* Common hogfenneI root*, and so forth.

## 4. Rethinking about the Case of Qinhu National Wetland Park

Traditional principals of urban water landscape planning do have limits nowadays considering the idea of orderly revetments, monotonous landscape forms, and plant arrangements. Only through breaking these limitations, can urban water landscape be innovated in terms of construction and transformation. It can be achieved through the following two aspects.

### 4.1. Macroaspects

*(1) Promotion and Education about the Value of Urban Water System.* Ignorance of urban water system for a long time has led to dramatic decrease in the number of water resources and poor water quality. Superficial understanding still exists in recent years, although growing consciousness has been seen on the protection of urban water system. Constructions with damage to water systems usually happen, especially in the process of building urban water landscape. Present community structure is the result of natural events and human intervention years by years [15]. Accordingly, it is necessary to inform and educate the public about their value and how to utilize them in a sustainable way. “Mass awareness programme” carried out by the government and NGOs may work on this aspect [16].

*(2) Coordinating the Utilization Plan of Urban Water System.* As an ecosystem, urban water system should be utilized in an integrated way in which different departments cooperate closely. It is an initial step to clarify how to use varied types of urban water resources, especially those located on the edge of plan areas so that they can be listed in green belt systems. People need to define the functions, attributes, and protecting scope of urban water and further compile protection and utilization plans in the guidance of city greenbelt plans.

*(3) Pollution Control to Protect Urban Water Ecology.* The sustainable utilization of urban water greatly relies on the improvements of water ecology. Therefore, it is a precondition to reduce and eradicate diversified sources of pollution which may bring destructive disaster for water ecology. At the same time, sewage closure is needed to avoid secondary contamination by separating rains and sewers.

*(4) Gradually Enhancing the Coverage and Quality of Urban Water.* If it is possible, artificial water landscapes can be built in cities accompanied with the reconstruction of water ecosystem. Through ecological techniques and engineering, degenerated or disappearing water system can be restored and reconstructed to provide the ecological functions of wetlands, as well as recreation areas for residents.

### 4.2. Microaspects

To some extent, the case of Qinhu wetland planning can be used for reference in urban water management in eastern China. According to the planning goals based on value and significance of city water resources, researches and production, environmental protection, leisure, and recreation should be combined so as to enrich users' experiences in water landscape and meanwhile enhance education effects. This evolutionary system needs to be in accordance with city development, the construction of ecological environment, and residents' demands in a sustainable principal. Urban water landscape planning should stress the following two parts.

*(1) Spatial Forms of Water Landscape.* Landscape ecology is focused on the relationship of spatial forms and ecological processes [17]. Researchers have been advocating the natural side of landscape, that is, to integrate “the spatially heterogeneous area” with its ecological functions [18]; for example, land-use activity is found to influence aquatic diversity [19], Ward et al. [18] proposed that patch size and shape contributed greatly on biodiversity, the research of Robinson et al. (2002) showed that the level of biodiversity decreased together with less ecotones, and Camacho-Valdez et al. (2013) and Chaikumbung et al. (2016) suggested that wetland characteristics, distribution, and contexts all had effect on their values. Generally speaking, structural complexity plays a positive role in ecosystem engineering [20]. A nonequilibrium state is common in ecosystems for their heterogeneity in time and space. Different water spatial forms can provide a plant friendly environment by increasing heterogeneity. It helps build a steady biome in the harmonious ecosystem. For example, natural biofilm can be made from large amounts of gravels in shoals and streams to purify suspended matters, nitrogen, phosphorus and heavy metals, and so forth. Plant roots, as well as the complex structure of soil, roots, and microorganism in the water, help degrade organic pollutants. Drops and waterfalls increase dissolved oxygen through the contact area of water and the air. However, urban water systems lack structural complexity and heterogeneity for “a smooth, engineered, almost vertical shoreline and bare substratum” [21]. In this case, diversified spatial forms of terrains and water should be fully utilized in water landscape planning.

*(2) Ecological Revetments.* Revetments play an important role in the material exchange between water and land. Therefore, water, land, and revetments should be taken as a whole to facilitate their interaction in revetment planning. Ecological planting is a primary part in revetments: waterfront plants and aquatic plants are connected to provide a sustainable natural environment for creatures through flat coastlines, natural transition from land to water, and coastline microtopography. Meanwhile, it leads the audience to change attention with the shape of coastlines. Varying sceneries with changing viewpoints can be achieved due to zigzagging natural banks. The functions of permeation and purification are given full play in ecological revetments in which the wetland increases its role of self-adjustment to bring a virtuous circle for the whole system, and at the same time, a vibrant landscape is enjoyed by the audience.

To sum up, in the improvement and innovation of urban water environment in eastern China, ecological landscape planning is needed to blend tradition with modernism. We should continually bring the latest scientific findings to existing methods to advance the development of water landscape planning in eastern China.

## Figures and Tables

**Table 1:** Sampling site conditions.

Locale	Longitude	Dimensionality	Area/ha	Dept/m	Lake and revetment form	Pollution level
Flora lake	31°59′23.84N	118°46′48.85 E	5.6	20	The lake is rectangular in shape, with a narrow distance in E-W direction. On average, its width is 200 m. Most of the revetments are in natural way.	IV

The Couple park	32°04′16.98N	118°48′22.69 E	10	1	The revetments are in natural way. The way the grassy slope extends into the water to make the revetments is used.	III

Xuanwu lake (Xuanwu men)	32°04′18.38N	118°47′25.84 E	378	1.4	The lake is rhombus in shape, with 15 km in circumference, 24 km in S-W direction and 2.0 km in E-W direction. The revetments are hard on the periphery and natural in the central island.	IV

Mochou lake	32°02′05.91N	118°45′19.36 E	0.37	1	The lake is triangle in shape, with 6 km in circumference. The revetments are mostly made of hard materials, such as rocks.	III

South lake park	32°01′51.08N	118°45′22.44 E	10	1.5	The lake is ellipse in shape. Its revetments are mostly made of soil or hard materials and natural revetments are very few.	IV

Yueya lake	32°01′57.66N	118°49′29.71 E	17.2	2	The lake is of North-South trend, with the water from Waiqinhuai river. The revetments, which are single in form, are made of hard materials.	III

Qinhuai lake-egret island park	32°01′16.09N	118°47′06.95 E	3.8	1.5	The lake is scattered, and the size of the lake is quite significant. The revetments consist of hard form mainly, with some natural forms such as the grass and rock.	IV

Dongshuiguan heritage (Qinhuai lake)	32°01′28.68N	118°47′37.57 E	1.55	1.1	The lake attaches to the Qinhuai river and its waterfront is hard revetment.	IV

Xianlin Lake	32°07′44.82N	118°58′41.28 E	18	2	The lake is just like a goose web, and the waterfront is in natural form which extends the grassland into water.	II

Yangshan park	32°06′24.76N	118°56′05.23 E	31	1.5	The lake makes a water circle by linking with Xihu park and Jiuxianghe wetland reserve. The revetment consists of hard form mostly.	III

Nanjing seven-bridge urn ecological wetland park	32°00′27.97N	118°50′08.58 E	1.2	1.7	The lake is rectangle, and the revetment is hardening by gravel and cement.	IV

Qianhu	32°03′00.63N	118°49′39.75 E	5.33	2	The size of the lake is just like a tadpole; the revetment was done in a natural way. Some of it is grassland-into-water, and some of it is in rock form.	IV

Zixia lake	32°03′41.50N	118°50′23.10 E	4.7	1.2	The surface is just like a block. Most of revetment is in artificial hard form with a little natural form.	III

Xiuqiu park	32°05′32.24N	118°44′20.71 E	3.6	2	The lake is trapezoid with plenty of hardening revetment and little rock form.	III

Foshou lake	32°06′18.00N	118°38′35.97 E	35	1.5	The lake is just like a hand, with hard and natural revetment combination.	II

**Table 2:** Suburban aquatic plants quantitative data.

Life style	Plant name	Frequency/%	Coverage/%	Important value	Serial number
Submerged plants	*Potamogeton crispus*	4	4.45	0.95	1
	*Ceratophyllum demersum*	5	0.21	0.05	2

Floating-leaved plants	*Spirogyra*	38	1.18	0.68	1
	*Lemna minor*	19	0.56	0.32	2

Hygrophytes	*Polygonum lapathifolium*	81	3.62	0.20	1
	*Ceratium glomeratum Thuill*	29	2.71	0.15	2
	*Ranunculus sceleratus*	57	2.36	0.13	3
	*Alternanthera Philoxeroides (Mart.) Griseb*	57	1.95	0.11	4
	*Veronica persica*	33	1.57	0.09	5
	*Lamium amplexicaule L.*	33	1.56	0.09	6
	*Ranunculus cuneifolius Maxim.*	43	0.93	0.05	7
	*Herba Ranunculi Japonici*	24	0.89	0.05	8
	*Cymbidium nanulum*	19	0.79	0.04	9
	*Be-gonia pedatifida Levl.*	14	0.52	0.03	10
	*Rorippa indica*	14	0.44	0.03	11
	*Mazus japonicus*	5	0.25	0.01	12
	*Poaannua*	5	0.13	0.01	13
	*Capsella bursa-pastoris (Linn.) Medic.*	10	0.07	0.01	14

Emerging plants	*Iris tectorum Maxim.*	38	3.37	0.41	1
	*Arundinella anomala Steud.*	38	2.69	0.32	2
	*Phragmites australis*	19	1.55	0.19	3
	*Typha orientalis*	5	0.43	0.05	4
	*Canna indica*	11	0.31	0.04	5

**Table 3:** Urban aquatic plants quantitative data.

Life style	Plant name	Frequency/%	Coverage/%	Important value	Serial number
Submerged plants	*Potamogeton crispus*	58.3	5.22	0.93	1
	*Ceratophyllum demersum*	12.5	0.40	0.07	2

Floating-leaved plants	*Spirogyra*	58.3	4.23	0.43	1
	*Lemna minor*	41.7	3.79	0.38	2
	*Nymphaea tetragona*	12.5	1.85	0.19	3

Hygrophytes	*Polygonum lapathifolium*	79.2	4.97	0.22	1
	*Alternanthera Philoxeroides (Mart.) Griseb*	66.7	4.57	0.19	2
	*Herba Ranunculi Japonici*	37.5	2.58	0.09	3
	*Ranunculus cuneifolius Maxim.*	29.2	2.25	0.07	4
	*Mimulus bodinieri Vant.*	25.0	1.46	0.04	5
	*Arundinella anomala Steud.*	12.5	1.23	0.04	6
	*Ranunculus natans*	20.8	1.08	0.05	7
	*Ranunculus sceleratus*	12.5	0.86	0.04	8
	*Marsilea L.*	4.2	0.83	0.04	9
	*Mazus japonicus*	12.5	0.79	0.02	10
	*Poaannua*	4.2	0.75	0.02	11
	*Capsella bursa-pastoris (Linn.) Medic.*	8.3	0.49	0.02	12
	*Veronica persica*	8.3	0.38	0.01	13
	*Cymbidium nanulum*	4.2	0.23	0.01	14
	*Trifolium repens*	4.2	0.20	0.01	15
	*Rorippa indica*	4.2	0.04	0.00	16

Emerging plants	*Iris tectorum Maxim.*	50.0	4.90	0.48	1
	*Phragmites australis*	63.4	4.79	0.34	2
	*Canna indica*	29.2	3.18	0.13	3
	*Typha orientalis*	12.5	0.99	0.06	4
	*Scirpus validus Vahl*	8.3	0.37	0.03	5
